# Electoral governance as a political determinant of health: state policy context, risk perception, and voter safety

**DOI:** 10.3389/fpubh.2026.1833040

**Published:** 2026-06-10

**Authors:** Selena E. Ortiz, Christopher Han-Fai Seto

**Affiliations:** 1 Department of Health Policy and Administration, The Pennsylvania State University (PSU), University Park, PA, United States; 2 Department of Sociology, Purdue University, West Lafayette, IN, United States

**Keywords:** administrative capacity, COVID-19, electoral governance, local election officials, political determinants of health, risk perception, vote by mail policy, voter safety

## Abstract

**Background:**

Electoral governance is a critical political determinant of health, yet the institutional mechanisms shaping population risk remain understudied. During the 2020 U.S. general election, local officials were responsible for administering voting amid COVID 19 threats and within uneven state electoral structures. Understanding how these political institutions influence officials' perceptions of voter safety and related actions is essential for characterizing the political determinants of health.

**Methods:**

We analyzed data from an October 2020 national CivicPulse survey of 493 local officials linked with county and state level indicators. Multinomial and binary logistic regression models assessed how individual characteristics, local pandemic conditions, and state electoral institutions—measured through voting by mail accessibility—predicted perceived voter safety concerns and municipal risk mitigation measures.

**Results:**

State electoral institutions were the strongest predictors of heightened voter safety concern, surpassing individual and county-level factors. Although political affiliation influenced perceived risk, higher concern among officials robustly predicted whether municipalities implemented harm reducing measures.

**Conclusions:**

Political institutions governing electoral access substantially shape local officials' recognition of public health risks and their protective responses. Electoral governance should be considered a structural component of public health preparedness, with implications for strengthening institutional capacity and democratic resilience.

## 1 Introduction

Voting is a critical political determinant of health in the United States since it enables communities to elect policymakers willing to promote and enact health-benefitting policies as well as determine the fate of policies establishing conditions essential to achieving health equity (1). Through democratic participation, communities can support policies related to health care access, quality education, housing affordability, economic opportunity, safe working environments, and other life-sustaining resources–structural conditions that undergird population health and health equity (2–4). Since the distribution of power, wealth, and political control exerts influence on health outcomes and the social determinants of health, voting is one way in which U.S. citizens can challenge the inequitable distribution of these resources, while inequities in voting can reflect, reinforce, and exacerbate broader health disparities (5–7). Therefore, ensuring safe and accessible elections is not solely a democratic imperative, but also a significant priority for population health (8).

In response, public health scholarship is increasingly emphasizing the role of the political determinants of health (9, 10), which can be understood as the political processes, structures, and decisions that distribute power and resources (11). Political systems not only influence the administration of resources but also the institutional capacity to identify, prioritize, and mitigate, population-level health risks (11). Although electoral governance is a particularly salient political determinant–as voting systems are both politically constructed and directly consequential for population exposure to harm–it remains understudied. While prior work has focused primarily on partisanship and ideological polarization, there has been less attention toward institutional capacity and administrative context (11, 12)^1^. Though emerging scholarship suggests that governance arrangements shape the distribution of resources, as well as how risks are recognized, prioritized, and acted upon by public officials during public health emergencies (13), empirical evidence linking institutional policy environments to local officials' perceptions of risk remains limited, especially outside of traditional public health agencies.

The 2020 U.S. general election offers a critical opportunity to examine how electoral governance functions as a political determinant of health and influences population exposure to risk during public health emergencies. Conducted during the COVID-19 global pandemic, the election generated national concerns regarding viral exposure, long wait times, and limitations on voting options (14). While public discourse emphasized individual behavior (15), responsibility for ensuring safe voting conditions largely fell to state and local governments already operating within decentralized and fragmented public health and election administration systems (16–19). Local officials, in particular, were positioned as intermediate institutional actors who were tasked with translating state-level electoral policies into health-relevant practices affecting voter safety (16), under conditions of extreme uncertainty and institutional constraint. Evidence that collaborative local governance arrangements shape population health outcomes during crises (20) underscores the need to examine how state policy environments condition local officials' assessments of feasibility, responsibility, and risk. However, despite this evidence, little is known about which factors shaped local officials' perceptions of voter safety during the pandemic, or whether such perceptions influenced the adoption of protective measures.

Risk perception is a well-established precursor to protective action in public health behavior theory (21, 22); however, few studies have examined whether this relationship extends to non-health governmental actors, especially those operating in polarized political environments. Moreover, it remains unclear whether local officials' assessments of voter safety risk–and subsequent decisions to implement protective measures–were driven primarily by local COVID-19 case burden, individual political characteristics, or by political institutional environments governing election processes. Research suggests that institutional contexts may function as cognitive and organizational cues, shaping how decision makers interpret uncertainty and evaluate the need for protective action (23).

Building on this framework, the present study analyzes national survey data from U.S. local government officials to examine how state-level electoral policy environments are associated with local officials' perceptions of voter safety and whether these perceptions are linked to municipal adoption of protective measures during a public health emergency. Distinct from prior research centered on voters' individual behaviors and perceptions, this study's unique contribution lies in shifting analytic focus upstream to local government officials, examining how state electoral policy environments shape institutional risk recognition and the adoption of harm-reducing practices during a public health emergency. Moreover, by empirically distinguishing between the drivers of risk recognition and the predictors of harm-reducing action, our study advances understanding of electoral governance as a political determinant of health and extends governance and risk-perception scholarship beyond traditional public health settings.

## 2 Methods

Data from the October 2020 CivicPulse omnibus survey were used. CivicPulse is a non-profit organization that generates insights of and for local governments through national surveys of local officials (including elected and top appointed leaders serving in municipal, township, and county governments across the U.S.) (24). It maintains an updated contact list of 16 local government positions associated with all townships, municipalities, and counties throughout the U.S. with populations of 1,000 or more. A random sample of local officials are invited to participate in the survey via email, and the survey is administered via the Qualtrics survey platform. CivicPulse surveys consistently include representation of local officials across both urban and rural municipalities throughout nearly all 50 U.S. states (24).

Our analyses utilize two samples: (a) an individual-level sample of 493 local officials, each associated with a specific county or municipality, that completed the survey and had no missing data on relevant study variables, and (b) a municipal-level sample of 523 local officials, including some that were missing on individual covariates but answered questions relevant to the municipality-level analyses. Analyses based on the individual-level data utilize probability weights, constructed by CivicPulse using conventional raking procedures, to account for the oversampling of both urban and populated areas and to increase overall representativeness (25). The weights were constructed using Census data on population, urbanization, and college-education, as well as county-level presidential vote share.

### 2.1 Dependent variables

The individual-level outcome of interest is local officials' level of concern for voter safety during the 2020 election. Respondents were asked, “*How serious of a concern is voter safety in relation to COVID-19 in your community?”* with responses: “Not at all a concern,” “Minor concern,” “Moderate concern,” and “Serious concern.” Due to the infrequent selection of “Not at all a concern” (*n* = 36), we combined these responses with those responses coded as “Minor concern” to construct a three-level categorical measure of safety concern. Sensitivity tests conducted using distinct, non-combined categories yielded substantively similar results to those presented here (results available upon request).

The municipal-level outcome of interest is whether local measures were implemented to improve voter safety during the COVID-19 pandemic. Regarding local election safety measures, respondents were asked: “*Aside from what your state is requiring, what steps has your local government taken to address voter safety concerns for the fall 2020 general election?”* Respondents were able to select all applicable responses from a list of measures: (a) “Expand online voter registration”; (b) “Mail absentee ballots to all registered voters”; (c) “Extend deadlines to receive mail ballots”; (d) “Increase in-person voting locations”; (e) “Other”; (f) “None”; and (g) “Unsure.” Affirmative responses to the first five categories were used to construct binary indicators of the measures taken. Qualitative descriptions of the “Other” measures were used to filter out responses that were equivalent to “None” or “Unsure.” The municipality-level analyses use a binary measure of whether *any* local measures were taken (“Yes” = 1; “None reported” = 0).

### 2.2 Independent variables

As prior studies have shown, personal attributes (e.g., political affiliation, gender, religion, and race/ethnicity) can influence local officials' views and actions (26), especially in times of public health crises (18). We examine individual-level characteristics that could predict local officials' level of concern: (a) sex (male = 1, female = 0); (b) college educational attainment (1 = 4-year college degree, 0 = no 4-year college degree); (c) race and ethnicity (1 = non-Hispanic white, 0 = minority race/ethnicity); (d) years of government experience (four categories of 0–9, 10–19, 20–29, and >30 years; and (e) political party affiliation (Democrat, Independent/other, Republican).

We also examine area-level characteristics that could be associated with the outcomes of interest. At the county-level, we examine: (a) COVID-19 case rate (per 1,000) based on new cases between July 1, 2020–September 30, 2020; (b) percent of votes cast for Donald Trump in the 2016 presidential election; (c) percent of residents at least 65 years of age; (d) percent non-Hispanic Black residents (logged); (e) percent Hispanic residents (logged); (f) a disadvantage principal component index based on percent poverty, less than high school education, unemployment, crowding, rented housing, single parent housing, and households with no vehicle (27); and (g) population density (logged). All county-level characteristics were measured as continuous variables.

To measure state-level electoral policy environments, specifically institutional capacity and administrative context, we used the Brookings Institution's “Voting-by-Mail in a Pandemic Scorecard” (hereinafter “Brookings Score”), which evaluated states ahead of the 2020 election on multiple dimensions of voting by mail on a five-letter grading scale, including: voting eligibility and access, ballot availability, and administrative preparedness and capacity (28). Grades possible were “A” (indicating most accessible); “B,” “C,” “D,”; and “F” (indicating least accessible).^2^ For our analyses, categories “D” and “F” were combined due to small cell sizes (*n* = ~19 and *n* = 2, respectively). There are several reasons why we believe the Brookings Score is an appropriate proxy for state policy environments. First, it directly evaluates the accessibility of voting-by-mail systems by assessing the ease of requesting, completing, and submitting a mail-in ballot, which were essential components of voter safety and access during the 2020 pandemic. Second, it reflects meaningful variation across states and distinguishes between states with highly accessible voting systems and states with limited or restrictive voting processes. Third, it captures aspects of preparedness and administrative capacity relevant to harm reduction strategies. Finally, it is externally developed and methodologically transparent, both of which contribute to its use as an independent evaluative tool designed to evaluate states' readiness to conduct safe elections during the pandemic.

### 2.3 Analysis

We begin by examining the individual-, county-, and state-level predictors of *safety concern* among local officials. We estimate a series of multinomial logistic regression models^3^, predicting safety concern among local officials using the individual- and county-level predictors described above, as well as state-level Brookings Score. Independent variables predict the odds of being in a higher category of concern (moderate or serious), relative to the category of no/minor concern. These regression models use the individual survey weights, with standard errors clustered at the state-level.

Next, we explore predictors of *local responses* taken to mitigate voter safety risks as reported by local officials. We estimate a binary logistic regression model that uses local officials' level of concern, county-level predictors, and state-level Brookings Score to predict the probability that municipalities engaged in any of the responses described above. Again, standard errors are clustered at the state-level.

At both analytic stages, we utilize model prediction to visualize the effects of key predictors on outcome probabilities, which were generated from fully controlled models with covariates held at their means. An alpha level of *p* < 0.05 was used for all statistical two-tailed tests. All analyses were conducted using Stata Statistical Software, version 18.0.

## 3 Results

Table 1 includes descriptive statistics for all relevant variables in both analytic samples described in detail below. As shown, local officials in the sample are evenly divided across levels of safety concern, with roughly a third of respondents in each concern category. Local officials are also more male and non-Hispanic white compared to the general U.S. population, and almost two thirds have attained a four-year college degree. The sample is diverse in terms of years of government experience and political affiliation.

**Table 1:** Descriptive statistics of local officials from the 2020 CivicPulse omnibus survey and linked county- and state-level data.

Characteristics	Individual sample (*N* = 493, weighted)		Municipality sample (*N* = 523, unweighted)		Data source
	Mean	Std.error	Mean	Std.error	
Level of concern					*2020 CivicPulse Omnibus Survey*
None/minor	0.32	(0.039)			
Moderate	0.34	(0.018)			
Serious	0.34	(0.041)			
Sex					*2020 CivicPulse Omnibus Survey*
Female	0.31	(0.025)			
Male	0.69	(0.025)			
Education					*2020 CivicPulse Omnibus Survey*
No college degree	0.37	(0.035)			
College degree	0.63	(0.035)			
Race					*2020 CivicPulse Omnibus Survey*
Minoritized race	0.12	(0.018)			
Non-Hispanic white	0.88	(0.018)			
Years gov. experience					*2020 CivicPulse Omnibus Survey*
0–9	0.44	(0.032)			
10–19	0.23	(0.020)			
20–29	0.16	(0.026)			
30 or more	0.17	(0.020)			
Political ID					*2020 CivicPulse Omnibus Survey*
Republican	0.42	(0.034)			
Independent/other	0.28	(0.026)			
Democrat	0.30	(0.025)			
County-level sociodemographics					
COVID-19 case rate (per 1,000)	11.83	(1.232)	11.91	(1.153)	*The New York Times ^1^*
Percent 65 and older	17.90	(0.431)	17.13	(0.372)	*2015–2019 ACS 5-year estimates ^1^*
Percent non-Hispanic Black^2^	7.02	(1.181)	8.18	(1.224)	*2015–2019 ACS 5-year estimates ^1^*
Percent Hispanic^2^	9.08	(1.461)	9.94	(1.559)	*2015–2019 ACS 5-year estimates ^1^*
Disadvantage index	−0.39	(0.133)	−0.39	(0.129)	*2015–2019 ACS 5-year estimates ^1^*
Percent Republican votes, 2016	56.23	(1.263)	51.73	(1.186)	*MIT Election Lab*
Population density^2, 3^	417.87	(51.925)	598.09	(74.869)	*2015–2019 ACS 5-year estimates ^1^*
Brookings Score					*The Brookings Institution*
A	0.14	(0.057)	0.18	(0.068)	
B	0.50	(0.106)	0.46	(0.103)	
C	0.32	(0.099)	0.32	(0.098)	
D/F	0.04	(0.021)	0.04	(0.020)	

Standard errors are clustered by US state. ACS = American community survey.

^1^ Data accessed via Social explorer.

^2^ Logged in regression models.

^3^ People per square mile.

Turning next to the county- and state-level measures, these means tend to be similar between the individual (weighted) and municipal (unweighted) samples, as well as similar national averages (a comparison between our analytic samples and all U.S. counties with regard to the county-level variables is available upon request). Descriptive statistics for the state-level Brookings Score show that most municipalities in the samples fall in the middle grade categories (“B” or “C”) with fewer municipalities in the more extreme categories.

Table 2 shows estimated logit coefficients and 95% confidence intervals from multinomial logistic regression models predicting probability of local officials reporting “moderate” or “serious” concern about voter safety referenced to “no/minor concern.” Model 1 includes only individual-level predictors, while Model 2 adds county-level predictors, and Model 3 adds state-level Brookings Score. Per each of the models, political affiliation is a strong individual-level predictor of self-reported voter safety concern with statistically significant effects. For example, per Model 1, Democrats have 438% higher odds than Republicans of reporting moderate concern as opposed to no/minor concern, net of the other individual-level characteristics. In regard to reporting serious concern, the effect of political affiliation remains substantial as Democrats also have 11 times higher odds than Republicans to report serious concern vs. no/minor concern.

**Table 2:** Estimated odds ratios and 95% confidence intervals from multinomial logistic regression models predicting concern for voter safety among local officials^1^.

	Model 1		Model 2		Model 3	
Variable	Odds ratio	95% CI	Odds ratio	95% CI	Odds ratio	95% CI
Moderate concern vs. no/minor concern						
Sex (Ref. Female)						
Male	1.18	[0.57, 2.45]	1.21	[0.58, 2.5]	1.16	[0.55, 2.44]
Education (Ref. no college degree)						
College degree	1.39	[0.81, 2.38]	1.32	[0.74, 2.36]	1.35	[0.74, 2.46]
Race (Ref. minoritized race)						
Non-Hispanic white	0.93	[0.42, 2.05]	0.88	[0.36, 2.13]	0.91	[0.36, 2.29]
Years gov. experience (Ref. 0–9)						
10–19	1.43	[0.69, 2.96]	1.47	[0.73, 2.99]	1.63	[0.77, 3.47]
20–29	0.75	[0.35, 1.6]	0.73	[0.33, 1.62]	0.81	[0.38, 1.74]
30 or more	2.01†	[0.99, 4.06]	2.07†	[0.97, 4.39]	2.10†	[0.95, 4.65]
Political ID (Ref. Republican)						
Independent/other	1.96^*^	[1.08, 3.55]	2.05^*^	[1.11, 3.78]	1.99^*^	[1.1, 3.57]
Democrat	5.38^***^	[2.54, 11.42]	5.84^***^	[2.42, 14.08]	6.12^***^	[2.61, 14.36]
COVID-19 case rate			1.00	[0.95, 1.04]	0.97	[0.93, 1.02]
Percent 65 and older			1.04	[0.97, 1.11]	1.04	[0.97, 1.12]
Logged percent NHB			1.29	[0.88, 1.9]	1.24	[0.82, 1.87]
Logged percent Hispanic			1.01	[0.71, 1.42]	1.10	[0.74, 1.62]
Disadvantage index			.95	[0.71, 1.28]	0.97	[0.73, 1.29]
Percent republican votes, 2016			1.01	[0.99, 1.03]	1.00	[0.98, 1.03]
Logged population density			1.11	[0.85, 1.45]	1.07	[0.81, 1.4]
Brookings score (Ref. A)						
B					1.27	[0.51, 3.14]
C					3.11^*^	[1.12, 8.64]
D/F					2.16	[0.41, 11.56]
Serious concern vs. no/minor concern						
Sex (Ref. Female)						
Male	1.06	[0.56, 2.01]	1.14	[0.59, 2.19]	1.07	[0.54, 2.11]
Education (Ref. no college degree)						
College degree	0.72	[0.37, 1.41]	0.64	[0.31, 1.34]	0.69	[0.34, 1.39]
Race (Ref. minoritized race)						
Non-Hispanic white	0.59	[0.24, 1.42]	0.61	[0.23, 1.61]	0.63	[0.23, 1.73]
Years gov. experience (Ref. 0–9)						
10–19	1.74	[0.8, 3.78]	1.73	[0.78, 3.84]	1.89	[0.86, 4.15]
20–29	1.27	[0.56, 2.85]	1.07	[0.47, 2.45]	1.30	[0.6, 2.83]
30 or more	1.53	[0.68, 3.41]	1.37	[0.6, 3.16]	1.28	[0.56, 2.91]
Political ID (Ref. republican)						
Independent/other	2.67^*^	[1.16, 6.11]	2.48^*^	[1.07, 5.71]	2.24^*^	[1.03, 4.84]
Democrat	11.00^***^	[5.32, 22.72]	9.93^***^	[4.88, 20.19]	11.12^***^	[5.53, 22.36]
COVID-19 case rate			1.04	[0.98, 1.09]	1.00	[0.94, 1.05]
Percent 65 and older			1.11^**^	[1.04, 1.18]	1.11^**^	[1.04, 1.18]
Logged percent NHB			1.19	[0.77, 1.84]	0.98	[0.62, 1.55]
Logged percent Hispanic			0.69†	[0.46, 1.04]	1.02	[0.69, 1.49]
Disadvantage index			1.12	[0.8, 1.56]	1.19	[0.88, 1.61]
Percent Republican votes, 2016			.99	[0.96, 1.02]	0.98†	[0.95, 1]
Logged population density			1.22	[0.92, 1.62]	1.11	[0.84, 1.47]
Brookings score (Ref. A)						
B					6.33^***^	[2.12, 18.89]
C					17.17^***^	[5.7, 51.76]
D/F					26.72^***^	[4.33, 164.81]
**Pseudo R**^**2**^	**0.08**		**0.11**		**0.14**	

^1^Data Sources: October 2020 CivicPulse Omnibus Survey; *The New York Times*; 2015–2019 ACS 5-year estimates; MIT Election Lab; and The Brookings Institution's *Voting by Mail in a Pandemic State-by-State Scorecard*.

*N* = 493 local officials; SE clustered by US state.

^***^p < 0.001; ^**^p < 0.01; ^*^p < 0.05; †*p* < 0.10.

Model 2 adds county-level predictors. Holding constant other predictors, a unit-increase in county-level percent of residents 65 years of age and older is associated with 11% higher odds of serious concern vs. no/minor concern. The effects of individual political affiliation persist even after adjusting for the county-level measures.

Model 3 adds the state-level Brookings Score. As shown, the strongest areal predictor is state-level Brookings Score, i.e., poorer scores are consistently associated with higher probability of serious concern. For example, net of individual- and county-level predictors and compared to living in a state that scored “A,” location in a state that scored “D” or “F” is associated with more than 26 times higher odds of serious concern (vs. no/minor concern) among local officials.

**Figure 1:**
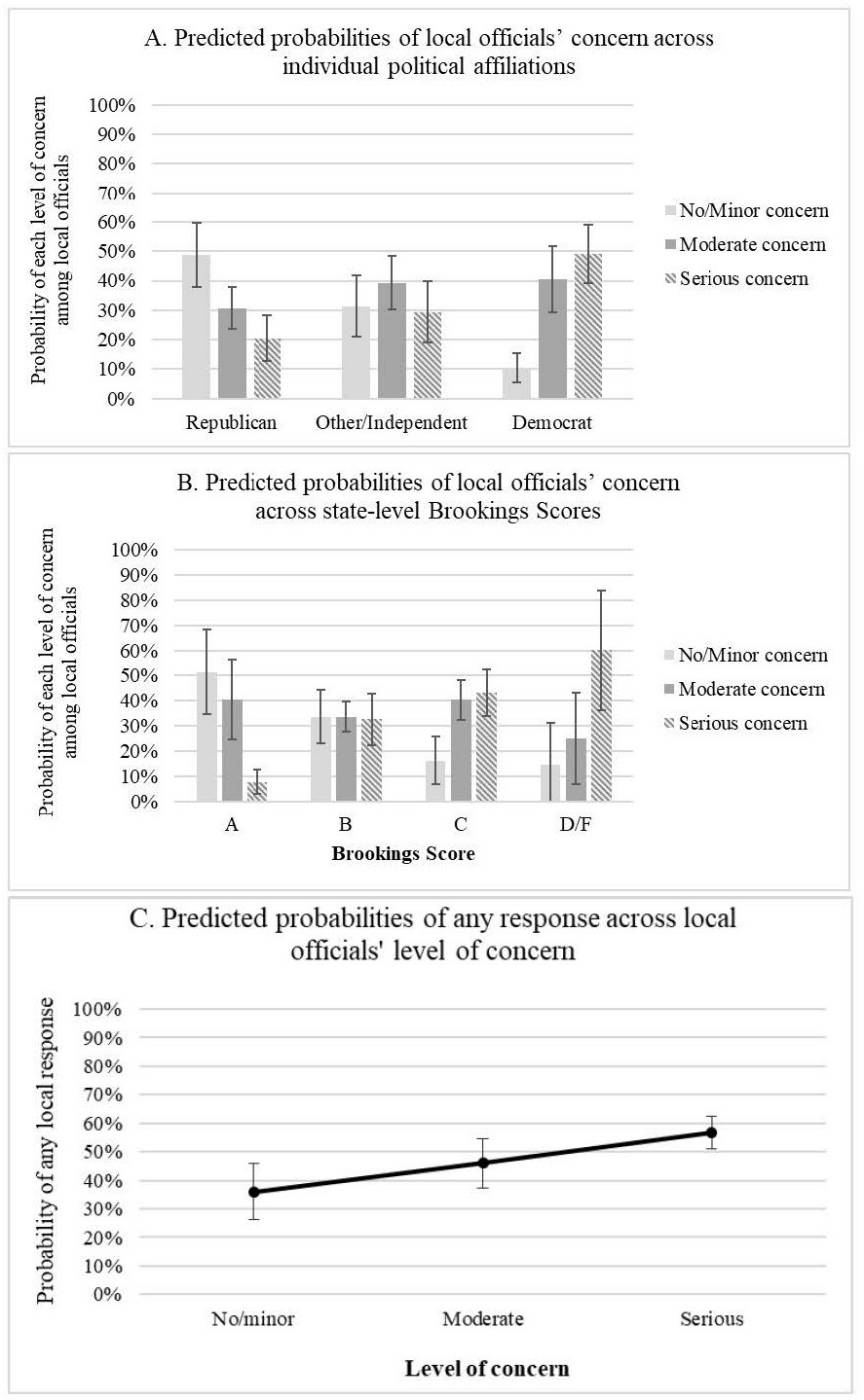
Predicted probabilities and 95% confidence intervals from multinomial and binary logistic regression models. Probabilities are model-based predictions and do not reflect crude prevalence. **(A)** Predicted probabilities of local officials concern across individual political affiliations. **(B)** Predicted probabilities of local officials concern across state-level Brookings Scores. **(C)** Predicted probabilities of any response across local officials level of concern.

Figures 1a, b show predicted probabilities of each level of concern across (a) individual political affiliation of local officials and (b) state-level Brookings Score associated with each municipality. The fully controlled model (i.e., Table 2, Model 3) was used to generate these predictions, and all other variables were fixed at their overall means. Per Figure 1a, there are stark differences in predicted concern for voter safety across individual political affiliations. For example, Republicans were predicted to have nearly a 50% probability of no/minor safety concern compared to only a roughly 20% probability of serious safety concern. In contrast, Democrats were predicted to have only a 10% probability of no/minor concern and almost a 50% probability of serious concern. Local officials who reported independent or other political affiliations had similar predicted probabilities across each level, with moderate concern being their most likely outcome.

Figure 1b shows similarly strong differences in predicted probabilities of concern across state groups defined by Brookings Score. As shown, local officials governing in states that received a grade of “A” had over a 50% predicted probability of no/minor concern and less than a 10% probability of serious concern. Conversely, local officials in states that received a grade of “D” or “F” had only about a 15% probability of no/minor concern and a 60% probability of serious concern.

Table 3 shows logit coefficients and standard error estimates from binary logistic regression models predicting whether a municipality engaged in any response to address voter safety (per the local official)^4^. As shown, the only significant predictor is the level of safety concern reported by that local official. Net of county- and state-level predictors and compared to municipalities where local officials reported no/minor safety concern, municipalities where local officials expressed serious concern had 132% higher odds of engaging in any response.

**Table 3:** Estimated logit coefficients and 95% confidence intervals from binary logistic regression models predicting local response^1^.

Variable	Any response	
	Odds ratio	95% CI
Safety concern (Ref. none/minor)		
Moderate	1.52†	[0.93, 2.48]
Serious	2.32^***^	[1.45, 3.74]
COVID-19 case rate	1.00	[0.97, 1.04]
Percent 65 and older	1.02	[0.98, 1.05]
Logged percent non-Hispanic Black	1.01	[0.76, 1.34]
Logged percent Hispanic	1.17	[0.83, 1.67]
Disadvantage index	1.00	[0.8, 1.25]
Percent Republican votes, 2016	0.99	[0.97, 1.01]
Logged population density	0.90	[0.74, 1.08]
Brooking score (Ref. A)		
B	1.48	[0.61, 3.58]
C	1.64	[0.75, 3.57]
D/F	1.02	[0.29, 3.53]

^1^Data Sources: October 2020 CivicPulse Omnibus Survey*; The New York Times*; 2015–2019 ACS 5-year estimates; MIT Election Lab; and The Brookings Institution's *Voting by Mail in a Pandemic State-by-State Scorecard*.

*N* = 523 municipalities; SE clustered by US state.

^***^p < 0.001; ^**^p < 0.01; ^*^p < 0.05; †*p* < 0.10.

Figure 1c shows predicted probabilities of engaging in a local response across reported level of concern. Adjusting for all county- and state-level predictors, municipalities where local officials reported no/minor concern had a 36% probability of adopting a local response. However, municipalities where local officials reported moderate concern or serious concern had a 46% and 57% probability, respectively, of adopting a local response.

## 4 Discussion

As with other perspectives about and responses to the COVID-19 pandemic, which were uneven and unequal across the U.S. (18), level of concern among local officials about the safety of the 2020 election process, as well as efforts to address these concerns, were mixed across municipalities. Our findings demonstrate that state-level electoral policy environments–captured by the Brookings Score–were the strongest predictors of perceived voter safety risk among local officials, even after adjusting for county-level partisanship and county-level COVID-19 burden. This finding suggests that political institutions governing electoral access serve as important political determinants of health, which is reinforced by evidence demonstrating that state policy environments are powerful predictors of population health outcomes (29, 30). Though the data reflect the 2020 election, we emphasize that the institutional dynamics identified here, particularly the role of state policy environments in shaping local risk recognition, are relevant as election administration continues to be contested and decentralized in the post-pandemic period (12, 16).

Furthermore, we found that a higher level of concern among local officials corresponded with greater probability of a reported local response to mitigate these concerns. These findings suggest that local officials' concerns not only parallel assessments demonstrating inability to ensure voter safety within their state but also serve as a contributing factor in the implementation of measures to safeguard voter wellbeing during election cycles. It could be, perhaps, that risk perception functions as a pathway through which the effect of political context on municipal action functions. These findings are reflective within public health behavior models and theories demonstrating how risk perception influences protective behavior, such as the Health Belief Model (HBM) (21). For example, within the HBM framework, the state-level Brookings Score can be understood as an institutional cue to action and signals administrative capacity and preparedness for voter safety, which shapes local officials' risk perception and motivates protective municipal decision making.

We also found that local officials' political affiliation strongly predicted perceived risk (Table 2). In contrast, municipal responses were modeled as a function of officials' expressed concern rather than individual partisanship (Table 3), with concern functioning as the proximal driver of public health-relevant interventions. Taken together, these findings suggest that although political identity influences whether risks are identified, action to mitigate risk is directly driven by risk perception itself. In this way, partisanship appears to determine upstream recognition of risk, rather than as a directly estimated determinant of municipal response. Therefore, health care partnerships with local policy leaders could be most effective when shared risk recognition is prioritized, vs. ideological views.

Beyond individual party affiliation, we found that no other individual characteristic was significantly associated with having either a moderate or serious level of concern for voter safety, compared to no/minor concern. Moreover, no other area-level indicator besides the Brookings Score was significantly associated with level of concern for voter safety. The exception was the percentage of adults aged 65 years or older, suggesting that local officials are sensitive to increased COVID-19 risk for senior voters.

In light of these findings, we consider why state vote-by-mail environments shape local officials' concern about voter safety. We raise three potential mechanisms: 1) *administrative and capacity signaling*; 2) *policy cues*; and 3) *resource anticipation*. First, it could be that vote-by-mail policies that were permissive and clearly articulated reduced perceived burden on local offices by establishing standardized processes, timelines, and contingency plans (28). Second, because vote-by-mail policies communicated priorities about voter access and safety, restrictive voting environments may have signaled limited institutional commitment to harm reduction, raising concern among local officials about overcrowding at polling places, masking, and service failures (14). Finally, state policies condition expectations about resources such as staffing, procedural flexibility, and access to physical and material resources (i.e., scarcity to personal protective equipment, sanitization supplies, and ability to modify polling sites), shaping perceived local capacity to mitigate identified risks (despite heightened risk perception) (16). Each of these mechanisms align with the pattern we observed: state vote-by-mail policy grades as captured by the Brookings' score strongly predicted serious concern, despite local COVID-19 burden, suggesting that institutional design operates as a cognitive and organizational cue for administrators.

To situate these findings within the broader evidence base, our study should be considered alongside other studies investigating how the COVID-19 pandemic shaped the administration of the 2020 U.S. election. For example, one study found that political party control of state legislatures was deterministic on the types of actions taken to address voter safety concern (12). The results emerging from our data, which offers a granular assessment of local perspectives and actions, also support the significance of partisan politics. However, our data also showed that local officials' level of safety concern–not party affiliation–was significantly associated with municipalities' reported adoption of efforts to minimize voter harm. This finding suggests the possibility of other factors as potential drivers of prosocial behavior to protect others. For example, another study found that consideration of other's health influenced initial support for the expansion of absentee ballots in Texas across the continuum of self-party identification (31). Future studies could examine how empathy for others could be invoked to increase and sustain support for policies that expand voter safety despite political affiliation. Our results also align with the U.S. Government Accountability Office (GAO) assessment of constrained election administration during the pandemic (16). Our study extends these findings by demonstrating that state legal frameworks and administrative capacity functioned as political determinants of health that shaped local officials' perceptions of voter safety risk.

### 4.1 Policy implications

Our findings carry immediate implications for election governance and public health preparedness. For *state election authorities*, vote-by-mail design is an upstream lever for risk recognition and harm reduction (28). Codifying clear eligibility rules, transparent timelines, and processing protocols can reduce administrative burden and stabilize operations during emergencies (16). For *local officials*, structured partnerships with health departments should be institutionalized before crises arise, which is consistent with research highlighting the importance of intergovernmental capacity during public health emergencies (17). In emergency contexts, such partnerships can facilitate real-time data sharing, including local disease transmission, outbreak alerts, and exposure risk, to support rapid assessment of voter safety risk and interagency coordination. Relatedly, for *public health agencies*, elections should be conceptualized and approached as recurring mass-gathering events that require routine surveillance and rapid technical assistance (8). Elections represent a nontraditional but consequential site of public health governance, particularly during public health emergencies. Since access to voting builds political and social capital that influences health status (32), voting systems and processes should be considered public-health relevant infrastructures, with design and management features that shape population exposure to harm. Framing elections this way strengthens the capacity to implement and sustain protections for voters, particularly those from marginalized communities. For *legislatures*, targeted investments in election infrastructure, such as mail-processing equipment or protective supplies, can strengthen local capacity when risk perception is already elevated among administrators (33). As future elections increasingly intersect with public health threats (e.g., infectious disease, climate-related emergencies, or infrastructure failures), integrating public health expertise into election administration is essential for safeguarding population health and promoting health equity.

### 4.2 Limitations

Our analyses are not without limitations. First, our data are cross-sectional and observational and lack measurement of the complex administrative processes by which concern among local public officials is translated into practice. For all of these reasons, causal interpretations of our findings should be made with caution. Extensions of this scholarship might benefit from the collection and analysis of similar data over time to improve causal identification. Second, though the use of national data and survey weights in model estimation improves sample representativeness, we rely on a relatively small sample of local officials for statistical inference. One consequence is diminished statistical precision; for example, the estimated odds ratios associated with levels of the Brookings Score in Table 2 had very wide confidence intervals, despite being statistically significant at conventional levels^5^. Furthermore, although our sample included officials from a wide range of contexts in terms of COVID-19 impact, with county-level case rates (per 1,000 people) ranging from 0.4 to 50.7 (and more than half of represented counties with case rates of more than 10 in our individual-level dataset), our ability to examine effect heterogeneity across geographic units or social context is somewhat limited. Future work could benefit from examining how racial/ethnic composition, socioeconomic disadvantage, and political diversity influences important links between election practices and public health. Third, because the data were collected during the 2020 election cycle, the data capture risk perception and decision-making under conditions of unusually high uncertainty and overstressed institutional capacity. Still, measuring voter safety perceptions contemporaneously during the election period helped avoid the recall bias that would affect *post-hoc* assessments of risk and decision making. Moreover, these findings are specific to the U.S. and should not be generalized to countries that administered elections under similar (or different) electoral governance structures during the COVID-19 pandemic. It would be beneficial for future research to examine the generalizability of our findings (i.e., whether similar patterns emerge in subsequent U.S. election cycles), as well as through comparative analyses in international settings. Fourth, self-reported perceptions of concern during a contentious election cycle could reflect political or social concerns. We encourage the use of longitudinal data and mixed methods study designs to better capture administrative processes and evolving institutional context in future work._Finally, although the instrument includes a survey item explicitly referencing voter safety *in relation to* COVID-19, some respondents may have interpreted the term “voter safety” differently, which could introduce some measurement imprecision.

## 5 Conclusion

Our study results demonstrate that state electoral policy environments shape local officials' perceptions of voter safety during public health crises, underscoring the importance of institutional design in shaping problem recognition within multilevel governance systems. By empirically distinguishing between the factors associated with risk perception and those linked to the adoption of harm-reducing measures, the findings highlight that governance contexts operate not only through formal policy mandates, but also through their influence on administrative cognition and decision-making.

As elections increasingly coincide with volatile health, environmental, and infrastructural threats, understanding how institutional environments influence local officials' assessments of risk becomes essential for strengthening policy responsiveness and effective public health preparedness. This study extends scholarship on the political determinants of health by demonstrating that electoral governance shapes exposure to harm not only through policies, but through upstream processes of risk recognition among non-health governmental actors. Strengthening institutional environments that support risk recognition among local officials may therefore represent an underutilized, but effective lever for reducing population exposure to harm during future public health emergencies.

## Data Availability

The raw data supporting the conclusions of this article will be made available by the authors, without undue reservation.

## Footnotes

1 In the context of electoral governance, we conceptualize institutional capacity as the legal authority, infrastructure, and procedural flexibility needed to administer elections under stress, while administrative context refers to the state policy environment that structures local administration through rules and signals of commitment to voter access and safety. In the decentralized U.S. system, state vote-by-mail policies are a central expression of both, for example.

2 Please see Appendix Table 1, which summarizes the criteria underlying the Brookings Institution's Voting-by-Mail in a Pandemic Scorecard.

3 Hausman tests conducted using the Stata *suest* command (on unweighted versions of the multinomial logistic regression models without clustered standard errors) suggested no violations of the assumption of the independence of irrelevant alternatives. Variance inflation factor (VIF) analysis yielded no VIFs higher than 4 for any of the models, suggesting no problematic extreme multicollinearity was present.

4 Table 3 models municipal responses as a function of officials' expressed concern and area-level characteristics, including county-level presidential vote share, rather than individual officials' political affiliations, which are examined only in the individual-level models predicting perceived risk (Table 2).

5 For example, the large, estimated effect for D/F states (OR = 26.72; 95% CI: 9.24–77.29) reflects limited data in this category, resulting in reduced precision and necessitating cautious interpretation.

## Appendix

**Table A1:** Key dimensions of the Brookings institution's voting-by-mail scorecard.

Dimension assessed	What it captures	Interpretation for voter safety
Eligibility and access	Who is legally permitted to vote by mail; ease of requesting a mail ballot	Broader eligibility and simpler request procedures reduce in-person congestion and exposure risk
Ballot availability and return	How ballots are distributed and returned (e.g., automatic mailing, drop boxes, deadlines)	Expanded ballot access and flexible return options facilitate physical distancing
Administrative preparedness and capacity	Clarity of rules, processing infrastructure, and ability to handle high mail-ballot volume	Greater preparedness signals institutional capacity to implement health-protective election practices
Overall grade (A–F)	Composite evaluation across dimensions	Higher grades indicate safer, more reliable, and more accessible election administration during the pandemic

The Brookings Institution scorecard evaluated state vote-by-mail systems prior to the 2020 election using publicly documented laws, regulations, and administrative guidance. Grades reflect comparative readiness to conduct a safe election under pandemic conditions. Data available at: https://www.brookings.edu/articles/voting-by-mail-in-a-pandemic-a-state-by-state-scorecard/.
